# Temporal Characterization of Microglia/Macrophage Phenotypes in a Mouse Model of Neonatal Hypoxic-Ischemic Brain Injury

**DOI:** 10.3389/fncel.2016.00286

**Published:** 2016-12-15

**Authors:** Nina Hellström Erkenstam, Peter L. P. Smith, Bobbi Fleiss, Syam Nair, Pernilla Svedin, Wei Wang, Martina Boström, Pierre Gressens, Henrik Hagberg, Kelly L. Brown, Karin Sävman, Carina Mallard

**Affiliations:** ^1^Institute of Neuroscience and Physiology, University of Gothenburg, Sahlgrenska AcademyGothenburg, Sweden; ^2^Centre for the Developing Brain, Perinatal Imaging and Health, King's College London, St. Thomas' HospitalLondon, UK; ^3^PROTECT, INSERM, Université Paris Diderot, Sorbonne Paris CitéParis, France; ^4^Department of Pediatrics, Institute of Clinical Sciences, University of Gothenburg, Sahlgrenska AcademyGothenburg, Sweden; ^5^Department of Obstetrics and Gynecology, Perinatal Center, Institute of Clinical Sciences, University of Gothenburg, Sahlgrenska AcademyGothenburg, Sweden; ^6^Department of Pediatrics, University of British Columbia and the Child and Family Research InstituteVancouver, BC, Canada

**Keywords:** perinatal brain injury, microglia, galectin-3, neuroinflammation

## Abstract

Immune cells display a high degree of phenotypic plasticity, which may facilitate their participation in both the progression and resolution of injury-induced inflammation. The purpose of this study was to investigate the temporal expression of genes associated with classical and alternative polarization phenotypes described for macrophages and to identify related cell populations in the brain following neonatal hypoxia-ischemia (HI). HI was induced in 9-day old mice and brain tissue was collected up to 7 days post-insult to investigate expression of genes associated with macrophage activation. Using cell-markers, CD86 (classic activation) and CD206 (alternative activation), we assessed temporal changes of CD11b^+^ cell populations in the brain and studied the protein expression of the immunomodulatory factor galectin-3 in these cells. HI induced a rapid regulation (6 h) of genes associated with both classical and alternative polarization phenotypes in the injured hemisphere. FACS analysis showed a marked increase in the number of CD11b^+^CD86^+^ cells at 24 h after HI (+3667%), which was coupled with a relative suppression of CD11b^+^CD206^+^ cells and cells that did not express neither CD86 nor CD206. The CD11b^+^CD206^+^ population was mixed with some cells also expressing CD86. Confocal microscopy confirmed that a subset of cells expressed both CD86 and CD206, particularly in injured gray and white matter. Protein concentration of galectin-3 was markedly increased mainly in the cell population lacking CD86 or CD206 in the injured hemisphere. These cells were predominantly resident microglia as very few galectin-3 positive cells co-localized with infiltrating myeloid cells in *Lys*-EGFP-*ki* mice after HI. In summary, HI was characterized by an early mixed gene response, but with a large expansion of mainly the CD86 positive population during the first day. However, the injured hemisphere also contained a subset of cells expressing both CD86 and CD206 and a large population that expressed neither activation marker CD86 nor CD206. Interestingly, these cells expressed the highest levels of galectin-3 and were found to be predominantly resident microglia. Galectin-3 is a protein involved in chemotaxis and macrophage polarization suggesting a novel role in cell infiltration and immunomodulation for this cell population after neonatal injury.

## Introduction

Hypoxic-ischemic (HI) brain injury is an important contributor to neonatal mortality as well as permanent neurological impairments in surviving infants. HI triggers an imbalance of CNS homeostasis and initiates peripheral and central inflammatory responses, which can be detected within 2–3 h of insult in rodent models (Hedtjärn et al., 2004; Bonestroo et al., 2013). Persistence of inflammation in the injured human infant brain is poorly defined, but hypothesized to continue for weeks to years (Fleiss and Gressens, 2012) contributing significantly to neurological outcome (Hagberg et al., 2015). Indeed, altering or reducing inflammation in the context of perinatal brain injury may have beneficial effects such as reducing lesion size (Hedtjärn et al., 2002; Kigerl et al., 2009; Bolouri et al., 2014).

Microglia are the primary immune competent and phagocytic cells of the brain (Kreutzberg, 1996). Despite ontogenetic dissimilarities (Ginhoux et al., 2010), microglia are broadly viewed as CNS counterparts to peripheral monocytes and macrophages. Experimental evidence from adult models show that brain injury rapidly activates microglia and lead to increased phagocytic activity and altered production of cytokines and reactive oxygen metabolites (Hanisch, 2002), features that are also well documented in neonatal HI (Hedtjärn et al., 2004). In the adult brain there is also a considerable contribution of infiltrating peripheral immune cells to the brain after stroke-like injury (Iadecola and Anrather, 2011). In contrast, little infiltration of peripheral cells is seen acutely after neonatal stroke (Denker et al., 2007), however, it remains unclear to what extent peripheral immune cells contribute to the inflammatory response after neonatal hypoxia-ischemia (Mallard and Vexler, 2015).

Early studies identified cytokines capable of inducing pro-inflammatory (classical) or anti-inflammatory (alternative) activities in macrophage cultures. Classically activated macrophages are commonly associated with the expression of surface antigen cluster of differentiation (CD) 86 and the expression of inducible nitric oxide synthase (iNOS) and pro-inflammatory cytokines including interleukin (IL) 1 and tumor necrosis factor alpha (TNF-α). Alternatively activated cells instead express CD206 and arginase 1 and have an enhanced production of anti-inflammatory cytokines (e.g., IL-4 and IL-10) and factors facilitating resolution of inflammation, immunomodulation, angiogenesis, and wound healing (Mantovani et al., 2004). Similarly, polarized pro- and anti-inflammatory phenotypes were demonstrated in cultured microglia in response to specific cytokine stimuli (Chhor et al., 2012). However, microglia phenotype expression patterns are age and region dependent (Scheffel et al., 2012; Grabert et al., 2016) and recent studies suggest a considerable overlap and complex pattern of activation states (Murray et al., 2014), which may be particularly apparent *in vivo*.

Galectin-3, a β-galactoside-binding lectin, is important for the regulation of alternative activation in macrophages (MacKinnon et al., 2008) and its expression is induced in microglia by anti-inflammatory cytokines (IL-4/IL-10) and repressed in response to pro-inflammatory stimulation (LPS) *in vitro* (Chhor et al., 2013). Microglia express galectin-3 after ischemic injury in adult and neonatal brain (Walther et al., 2000; Doverhag et al., 2010) and in the adult brain galectin-3 is associated with protective IGF-1-expressing microglia after stroke (Lalancette-Hébert et al., 2007). However, galectin-3 is also a strong chemoattractant for monocytic cells (Sano et al., 2000), induces production of pro-inflammatory cytokines and we have previously demonstrated that galectin-3 contributes to neonatal HI injury (Doverhag et al., 2010). Galectin-3 is thus of specific interest in the polarization and modulation of microglia phenotypes following HI injury.

In this study we induced HI in postnatal day (P) 9 mouse pups, an age equivalent to the near term human infant with respect to brain developmental stage (Craig et al., 2003). We investigated the temporal expression of genes previously associated *in vitro* with classical and alternative polarization phenotypes and used well-defined macrophage cell-surface CD antigens to identify specific phenotypes within the CD11b^+^ population (general microglia/macrophage marker) in the brain following neonatal HI. Finally, to explore the role of the immunomodulatory factor galectin-3 in polarization of CD11b^+^ cells after HI, we characterized the expression of galectin-3 in different post-HI cell populations in the brain.

## Materials and methods

### Animals

Pregnant C57BL/6 mice were sourced from Charles River Laboratories International (Sulzfeld, Germany). *Lys*-enhanced green fluorescent protein (EGFP)-*ki* mice were obtained from Dr. Tomas Graf, Autonomous University of Barcelona. Animals were housed at the Laboratory for Experimental Biomedicine at University of Gothenburg under specific pathogen free conditions on a 12 h light/dark cycle with *ad libitum* access to standard laboratory chow (B&K, Solna, Sweden) and water.

### Hypoxic-ischemic brain injury model

Hypoxic-ischemic (HI) brain injury was induced in P9 mice (of both sexes) based on methods developed by Rice et al. (1981), with some modifications for mice (Doverhag et al., 2010). In brief, mice were anesthetized with isoflurane in a 1:1 oxygen and nitrous oxide mix and the left common carotid artery was permanently ligated with a 6-0 prolene suture. Mice were returned to their home cage for 1 h of recovery and then transferred to a temperature regulated incubator for a 50 min period of hypoxia (36°C, 10% O_2_). The HI insult result in injury in the ipsilateral (ipsi) hemisphere, typically in the cortex, hippocampus and striatum, while there is no morphological injury in the contralateral (contra) hemisphere as previously reported by our group (Svedin et al., 2007). Sham-operated animals were not exposed to artery ligation and hypoxia.

### Reverse transcription and qRT-PCR

Mice were deeply anesthetized and transcardially perfused with ice-cold 0.9% saline. Brains were rapidly removed, hemispheres separated, and snap-frozen on dry ice before being stored at −80°C. Total RNA was isolated using an RNeasy Lipid Tissue Mini Kit (Qiagen, Sollentunna, SE) in accordance with the manufacturer's instructions. RNA concentration was measured using a NanoDrop 1000 spectrophotometer (NanoDrop, Wilmington, USA) and RNA quality was determined by Experion Chip RNA analysis (BioRad, Solna, SE) (RQI value 8–10 for all samples). Reverse transcription was performed in duplicate using a QuantiTect Reverse Transcription Kit (Qiagen). qRT-PCR was performed on a Roche LightCycler480 (Roche, Bromma, SE) using a QuantiFast SYBR Green PCR kit (Qiagen) with the following cycling protocol: 10 s denaturation at 95°C followed by 30 s annealing/extension at 60°C for 40 cycles. All primers were purchased from Qiagen (Table 1) and amplification specificity was confirmed by melting curve analysis. Relative quantitation was performed in accordance with the standard curve method and expression values were normalized to the reference gene glyceraldehyde 3-phosphate dehydrogenase (*GAPDH*).

**Table 1:** **Primers used for qRT-PCR**.

**Gene**	**Official symbol**	**Entrez gene ID**	**Primer**	**Product number**
Gapdh	Gapdh	14,433	Mm_Gapdh_3_SG	QT01658692
CD86	Cd86	12,524	Mm_Cd86_1_SG	QT01055250
IL1b	Il1b	16,176	Mm_Il1b_2_SG	QT01048355
Cox2	Ptgs2	19,225	Mm_Ptgs2_1_SG	QT00165347
iNos	LOC673161	673,161	Mm_LOC673161_1_SG	QT01547980
CD206	Mrc1	17,533	Mm_Mrc1_1_SG	QT00103012
IL10	Il10	16,153	Mm_Il10_1_SG	QT00106169
Fizz1	Retnla	57,262	Mm_Retnla_1_SG	QT00254359
Arg1	Arg1	11,846	Mm_Arg1_1_SG	QT00134288
Gal3	Lgals3	16,854	Mm_ Lgals3_1_SG	QT00152558
IL6	Il6	16,193	Mm_Il6_1_SG	QT00098875

### Immunohistochemistry

Animals were deeply anesthetized and transcardially perfused with ice-cold 0.9% saline followed by buffered 6% formaldehyde (Histofix; Histolab, Gothenburg, Sweden). Brains were rapidly removed, post-fixed for 24 h at 4°C, cryoprotected in 30% sucrose, and sectioned at 25 μm on a Leica CM3050 S cryostat (Leica, Sweden). Non-specific antibody binding sites were blocked through a 30 min room temperature incubation in TBS containing 3% donkey serum and 0.1% Triton X-100 (hereafter referred to as blocking buffer). Sections were then incubated overnight at 4°C with the following primary antibodies diluted in blocking buffer: rabbit anti-ionized calcium binding adapter molecule 1 (Iba1) (1:1000, cat. no 019-19741, Wako, Osaka, JP), rat anti-mouse CD86 (1:200, cat. no. 14-0862, eBioscience, San Diego, USA), goat anti-CD206 (1:200, cat. no. AF2535, R&D Systems, Abingdon, UK), chicken anti-galectin-3 (1:1000, gift from Prof. Anna Karlsson, University of Gothenburg) and rat anti-galectin-3 (1:100, gift from Prof. Anna Karlsson, University of Gothenburg) and rabbit anti-GFP (1:1000, cat. no. ab290, Abcam, Cambridge, UK). Secondary antibodies were diluted 1:1000 in blocking buffer and applied for 2 h at room temperature: donkey anti-chicken CF488 (cat. no. SAB4600031, Sigma-Aldrich, Saint Louis, MO, USA), donkey anti-rat Cy3 (cat. no. 712-165-153, Jackson, Newmarket, UK), donkey anti-goat Alexa Fluor 633 (A-21082, Life Technologies, Sweden), donkey anti-rabbit Alexa Fluor 647 (A-31573, Life Technologies, Carlsbad, CA, USA), donkey anti-rat Alexa Fluor 488 (A-21208, Life Technologies), donkey anti-rat Alexa Fluor 594 (A-21209, Life Technologies) and donkey anti-rabbit Alexa Fluor 488 (A-21206, Life Technologies).

Images were captured on a Zeiss Laser Scanning 700 inverted confocal microscope equipped with Zen Black control software (Zeiss, Oberkochen, DE) and processed using Velocity (Perkin-Elmer). Figures were compiled using Adobe CS6 (Adobe, Kist, SE).

### Fluorescence-activated cell sorting (FACS)

Sham-operated and HI animals were deeply anesthetized and sacrificed via decapitation at 24 h, 72 h, and 7 days after surgery. Brains were rapidly removed, hemispheres separated, and transferred to ice-cold Hibernate-A (Invitrogen). In order to generate sufficient material for downstream analysis, corresponding hemispheres from two animals were pooled. Single cells suspensions were obtained through enzymatic dissociation in Ca^2+^ and Mg^2+^ free Hank's Balanced Salt Solution (HBSS) containing 0.01% papain (Bionordika, Stockholm, SE), 0.1% Dispase II (Bionordika), 0.01% DNase I (Roche, Bromma, SE) and 12.4 mM MgSO_4_. Briefly, samples were coarsely chopped and further dissociated through three rounds of enzymatic (37°C, 10 min) and mechanical digestion (repeated pipetting) before addition of 10 ml of warm Hibernate-A and filtration over a 40 μm cell strainer. Single cell suspensions were then quantified on a BioRad TC10 automated cell counter (BioRad, Solna, SE), centrifuged (350 g, 5 min), supernatant discarded, resuspended in 1 ml of staining buffer [0.5% bovine serum albumin (BSA), 2 mM ethylenediaminetetraacetic acid (EDTA), 0.09% Sodium Azide in phosphate buffered saline (PBS)], and incubated at 4°C for 20 min with the following primary antibodies: PerCP/Cy5.5 anti-mouse CD86 antibody (1 μg/10^6^ cells, cat. no 105028, BioLegend), APC anti-mouse CD206 antibody (0.5 μg/10^6^ cells, cat. no 141708, BioLegend) and PE/Cy7 anti-mouse/human CD11b antibody (0.25 μg/10^6^ cells, cat no 101216, BioLegend). Stained samples were supplemented with 10 ml staining buffer, pelleted (350 g, 5 min), re-suspended in 500 μl staining buffer, and kept on ice until FACS sorting.

Cells were sorted on a FACS Aria II Cell Sorter (BectonDickinson) equipped with a 100 μm nozzle. Primary gates were established on forward/side scatter plots (**Figure 2A**) using appropriate isotype controls. Cells positive for CD11b (**Figure 2B**) were further analyzed for their expression of CD86 and CD206 in the contralateral non-injured (**Figure 2C**) and ipsilateral injured hemisphere (**Figure 2D**). Gating strategies were based on previous studies (Bedi et al., 2013). Total cell number per sorted population was recorded and representative data was captured for the first 20,000 cells. Following sorting, cells were pelleted (1000 g, 10 min), immediately frozen on dry ice and stored at −80°C until cell lysis/protein analysis.

### Galectin-3 protein analysis

Cell pellets were lysed using a Bio-Plex Cell Lysis Kit (BioRad) in accordance with manufacturer's instructions. Briefly, lysing solution was added and cells resuspended by repeated pipetting before agitation on a microplate shaker (150 rpm, 4°C, 15 min). Samples were then centrifuged (4000 g, 10 min) and supernatants collected and stored at −20°C. Protein concentration was measured using a Pierce BCA Protein Assay Kit (Thermo Scientific) as outlined in the manufacturer's instructions. Galectin-3 protein was measured using a Galectin-3 enzyme-linked immunosorbent assay (ELISA) Kit (cat. no 12727, BG Medicine). Data is presented as total amount target protein per ml (pg/ml) or as normalized to protein concentration and presented as pg target protein per μg total loaded protein. Sample values falling below the lower limit of detection were substituted with the manufacturer stated lower detection limit divided by the square root of 2.

### Statistical analyses

All data are presented as group mean ± SEM. Data from males and females were combined in each group as breakdown by sex revealed no sex-specific expression of any of the markers analyzed. qRT-PCR and FACS data were assessed by two-way analysis of variance (ANOVA) followed by Tukey's multiple comparisons test. Galectin-3 protein concentrations over time were assessed by Kruskal-Wallis followed by Dunn's multiple comparison test for each cell population vs. sham. Differences were considered significant at ^*^p ≤ 0.05; ^**^p ≤ 0.01; ^***^p ≤ 0.001. Analyses were performed using Prism (Graphpad, v.6.05)

## Results

### Neonatal HI induces rapid expression of genes associated with classical and Alternative activation

**Figure 1:**
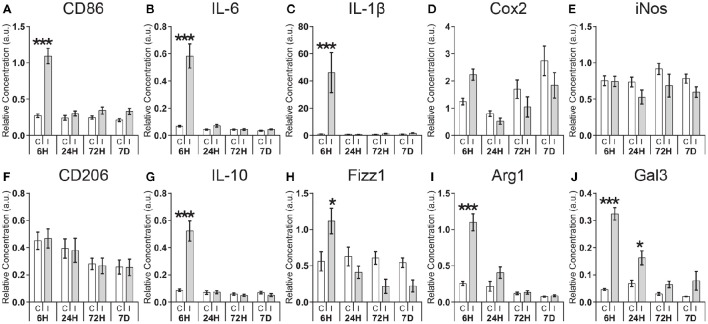
**Expression of pro- and anti-inflammatory genes following neonatal hypoxia-ischemia**. Expression of genes associated with classic activation (*CD86, IL-6, IL-1*β*, Cox2, iNOS;* ***A–E***), alternative activation (*CD206, IL-10, Fizz1, Arg1;* ***F–I***), and immunomodulatory (*Gal3;* ***J***) was measured by qRT-PCR at 6, 24, 72 h, and 7 days in the contralateral **(C)** and ipsilateral **(I)** hemisphere after HI. Values are presented as mean ± SEM. Analysis by two-way ANOVA followed by Tukey's multiple comparisons test between ipsilateral and contralateral hemispheres; *n* ≥ 10 per group; ^*^p ≤ 0.05, ^***^p ≤ 0.001.

Using total cortical homogenates we performed a qRT-PCR assessment of temporal expression profiles of genes associated with classical activation (*CD86, IL-6, IL-1*β*, Cox2, iNOS*) and alternative activation (*CD206, IL-10, Fizz1, Arg1*) in macrophages, as well as the immunomodulatory factor galectin-3 (*Gal3*) following HI (Figure 1). For all genes examined there was a significant interaction between expression in the ipsi- or contralateral hemisphere and with time, except for *CD206* and *iNos* (Supplementary Table 1). *Post-hoc* analysis revealed acute regulation in the ipsilateral hemisphere compared with the contralateral hemisphere of genes associated with classical activation: *CD86* (4.0-fold; *p* < 0.001), *IL-6* (8.6-fold; *p* < 0.001), *IL-1*β (46.3-fold; *p* < 0.001); as well as genes associated with alternative activation: *IL-10* (6.0-fold; *p* < 0.001), *Fizz1* (2.1-fold; *p* < 0.001), *Arg1* (4.3-fold; *p* < 0.001) at 6 h after HI, with no significant regulation at later time points. Enhanced expression of the immunomodulatory gene *Gal3* could be detected at 6 h (7.0-fold; *p* < 0.001) and 24 h (2.4-fold; *p* < 0.05) after HI. The genes *Cox2, iNos*, and *CD206* displayed no significant differences between contralateral and ipsilateral hemispheres at any of the time points investigated.

### Different CD11b^+^ cell subpopulations are present in the injured hemisphere after hypoxia-ischemia

**Figure 2:**
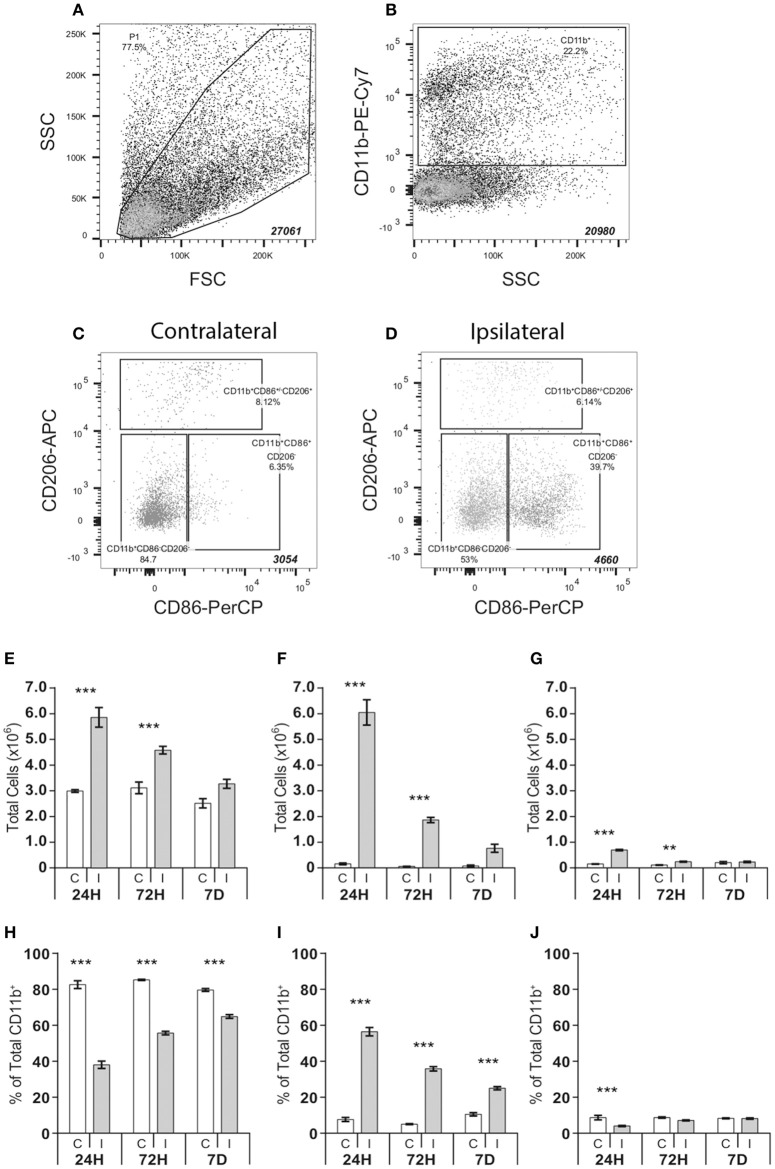
**FACS analysis of CD11b positive cell populations in the brain after hypoxia-ischemia**. Microglia cell populations were quantified in single cell suspensions isolated from ipsilateral and contralateral cerebral hemispheres following hypoxia-ischemia. Samples were progressively gated based first on size (FSC) and granularity (SSC) **(A)** followed by CD11b **(B)** and finally CD86 and CD206 (C-D) immunoreactivity, allowing identification of three distinct cell populations: CD11b^+^CD86^−^CD206^−^, CD11b^+^CD86^+^CD206^−^ and CD11b^+^CD86^+^/^−^CD206^+^ **(C,D)**. Absolute number and % of total number of CD11^+^ cells of CD11b^+^CD86^−^CD206^−^**(E,H)**, CD11b^+^CD86^+^CD206^−^ **(F,I)** and CD11b^+^CD86^+^/^−^CD206^+^ **(G,J**) cell populations in contralateral **(C)** and ipsilateral **(I)** hemispheres at 24 h, 72 h and 7 days after HI. Values presented as mean ± SEM. Analysis by two-way ANOVA followed by Tukey's multiple comparisons test between ipsilateral and contralateral hemispheres at each time point; *n* = 6–7 per group; ^**^p ≤ 0.01, ^***^p ≤ 0.001.

Having observed acutely elevated expression of inflammation-associated genes in the ipsilateral hemisphere, we next asked how the composition of microglia/macrophages in the brain may be regulated in response to HI. We employed FACS analysis to assess the total number of CD11b^+^ cells and to characterize cell phenotypes within this population at different time points after HI. Cells were characterized by a stepwise gating strategy based on methods described by Bedi et al. (2013). This facilitated identification of three major cell populations including classically polarized-like cells (CD11b^+^CD86^+^CD206^−^), alternatively polarized-like cells (CD11b^+^CD86^+/−^CD206^+^) and a cell population that did not express either CD86 or CD206 (CD11b^+^CD86^−^CD206^−^) (Figures 2A–D).

### Hypoxia-ischemia triggers expansion of the CD11b^+^ cells for up to 7 days

HI resulted in a significant expansion of the total number of CD11b^+^ cells, which was both time and hemisphere dependent (Supplementary Table 2), resulting in an increase of these cells in the ipsilateral hemisphere at 24 h (+282.4%; *p* < 0.001), 72 h (+103.4%; *p* < 0.001), and 7 days (+53.4%; *p* < 0.01). This effect was mirrored in the expansion of the CD11b^+^CD86^−^CD206^−^ cell population (24 h, +95.6%, *p* < 0.001; 72 h, +47.0%, *p* < 0.001) (Figure 2E), the CD11b^+^CD86^+^CD206^−^ cell population (24 h, +3667.1%, *p* < 0.001; 72 h, +2898.8% *p* < 0.001) (Figure 2F), and the CD11b^+^CD86^+/−^CD206^+^ cell population (24 h, +402.2%, *p* < 0.001 and 72 h, +138.2%, *p* < 0.001, Figure 2G).

There was an interaction between hemisphere and time after HI that determined the ratio of different cell subtypes (Supplementary Table 3). In the ipsilateral hemisphere, the CD11b^+^CD86^+^CD206^−^ population, in relative terms, was the dominant cell type at 24 h after HI constituting 56% of the total number of CD11b^+^ cells, compared to 7% in the contralateral hemisphere (ipsi 56.4 ± 2.4% vs. contra 7.2 ± 1.1%, *p* < 0.0001, Figure 2I). At 72 h post-injury, 35% of the CD11b^+^ cells in the ipsilateral hemisphere were CD86^+^CD206^−^, compared to 5% in the contralateral hemispheres (ipsi 35.1 ± 1.2% vs. contra 4.8 ± 0.3%, *p* < 0.0001). At 7 days post-HI, the percentage of this cell population in the ipsilateral hemisphere was reduced compared to levels at 24 h post-injury, yet remained 2.5-times higher compared to the contralateral hemisphere (ipsi 24.4 ± 0.9% vs. contra 10.0 ± 0.9%, *p* < 0.0001).

The proportion of CD11b^+^CD86^+/−^CD206^+^ cells never exceeded 8% of the total CD11b^+^ population, in either the contra- or ipsilateral hemisphere, at any time point examined. Despite an early increase in absolute numbers (Figure 2G), the percentage of CD11b^+^CD86^+/−^CD206^+^ cells was reduced in the ipsilateral compared to the contralateral hemisphere, at 24 h (ipsi 3.2 ± 0.2% vs. contra 5.7 ± 0.7%, *p* < 0.01, Figure 2J). By 3 days post-injury the CD11b^+^CD86^+/−^CD206^+^ population in the ipsilateral hemisphere had approximately assumed the same relative size as in the contralateral hemisphere (Figure 2J).

In absolute numbers the CD11b^+^CD86^−^CD206^−^cell population remained elevated over time in the ipsilateral compared to the contralateral hemisphere (Figure 2E). However, due to the large increase in CD11b^+^CD86^+^CD206^−^ cells in the ipsilateral hemisphere (Figure 2F), CD11b^+^CD86^−^CD206^−^ cells constituted a smaller proportion of the total CD11b^+^ population (Figure 2H) in the ipsilateral hemisphere compared with the contralateral at 24 h (ipsi 36.4 ± 4.8% vs. contra 81.4 ± 5.7%, *p* < 0.001), at 72 h (ipsi 54.7 ± 2.5% vs. contra 84.7 ± 1.0%, *p* < 0.001) and at 7 days (ipsi 63.9 ± 2.5% vs. contra 78.8 ± 2.1%, *p* < 0.001) post-injury.

The CD11b^+^CD86^−^CD206^−^ population represented the largest population in the contralateral hemisphere (approximately 80% of all CD11b^+^ cells (Figure 2H), where the cell number was relatively constant during the study period (24 h: 299,680 ± 5164; 72 h: 311,810 ± 22,950; 7 days: 251,673 ± 18,105) (Figure 2E).

### Identification of microglia/macrophage phenotypes by immunohistochemistry

**Figure 3:**
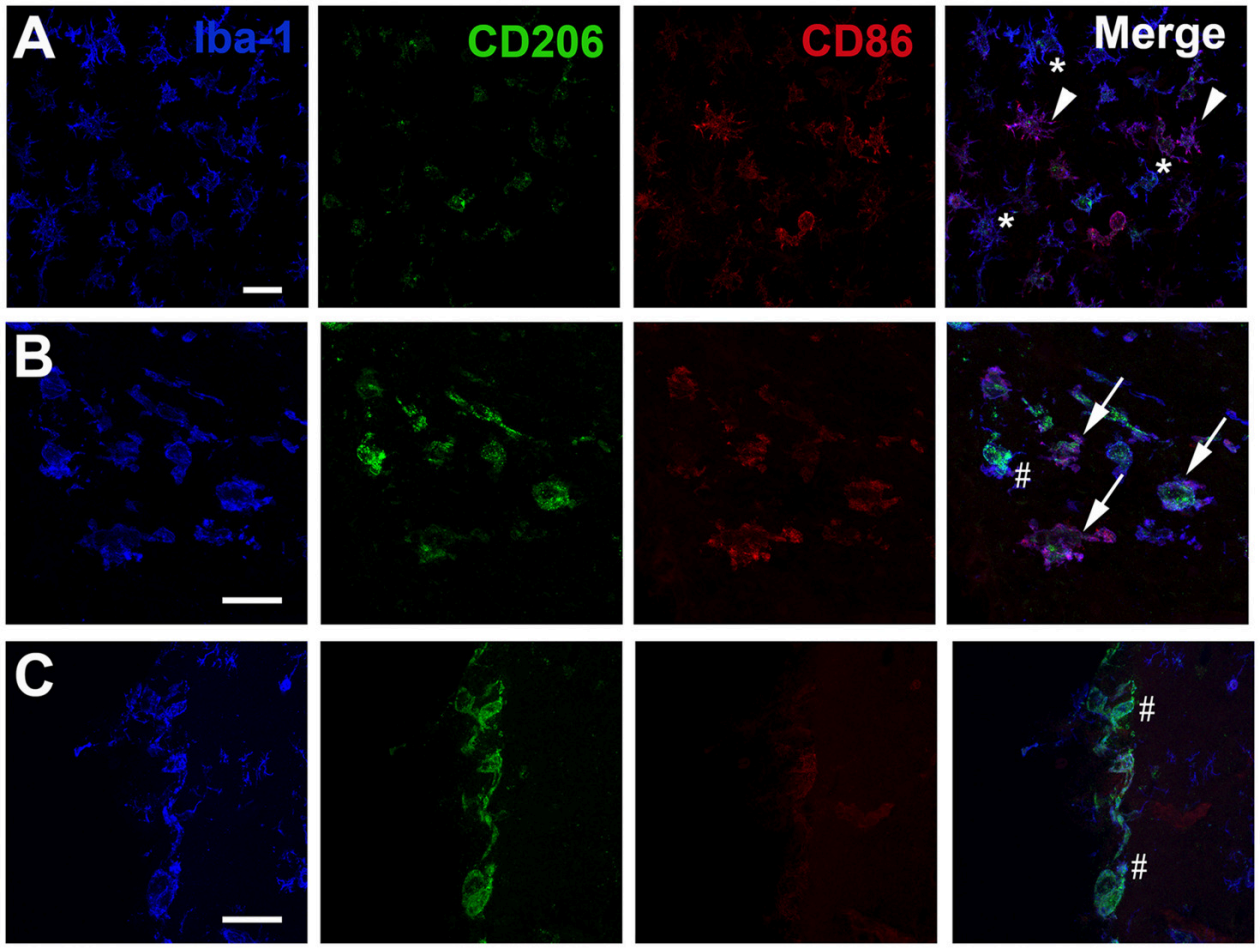
**Immunohistochemical visualization of CD86 and CD206 in Iba1 positive cells after neonatal hypoxia-ischemia**. Representative photomicrographs display immunoreactivity for Iba1, CD206, and CD86 in the striatum **(A)** the corpus callosum **(B)** and at the pial meningeal surface **(C)** of the ipsilateral hemisphere 24 h after hypoxia-ischemia. **(A)** One star indicates Iba1 positive cell lacking CD86/CD206 immunoreactivity; white arrowhead indicates Iba1 positive co-labeled with CD86. **(B)** White arrow indicates Iba1 cell positive for both CD86 and CD206 and hashtag identifies Iba1 positive cell which is mainly co-labeled with CD206. **(C)** Hashtag show Iba-1 positive cells only co-labeled with CD206. Scale bars: 25 μm.

The presence of different cell phenotypes was confirmed by confocal analysis on immuno-stained histological sections. Cells, expressing Iba-1 (microglia/macrophage marker), but neither CD86 nor CD206, were readily observed in injured areas (Figure 3A) as well as in the contralateral hemisphere (data not shown). Iba-1 positive cells with strong expression of CD86 were also found in the ipsilateral hemisphere (Figure 3A). In white matter areas, such as the corpus callosum (Figure 3B), Iba-1/CD206-positive cells were noted adjacent to Iba-1 positive cells clearly expressing both CD206 and CD86. Interestingly, Iba-1 positive cells expressing CD206 only, were mainly found in the meninges (Figure 3C) or the contralateral hemisphere (data not shown).

### Galectin-3 is primarily expressed in CD11b^+^ cells that lack CD86 and CD206 after hypoxia-ischemia

**Figure 4:**
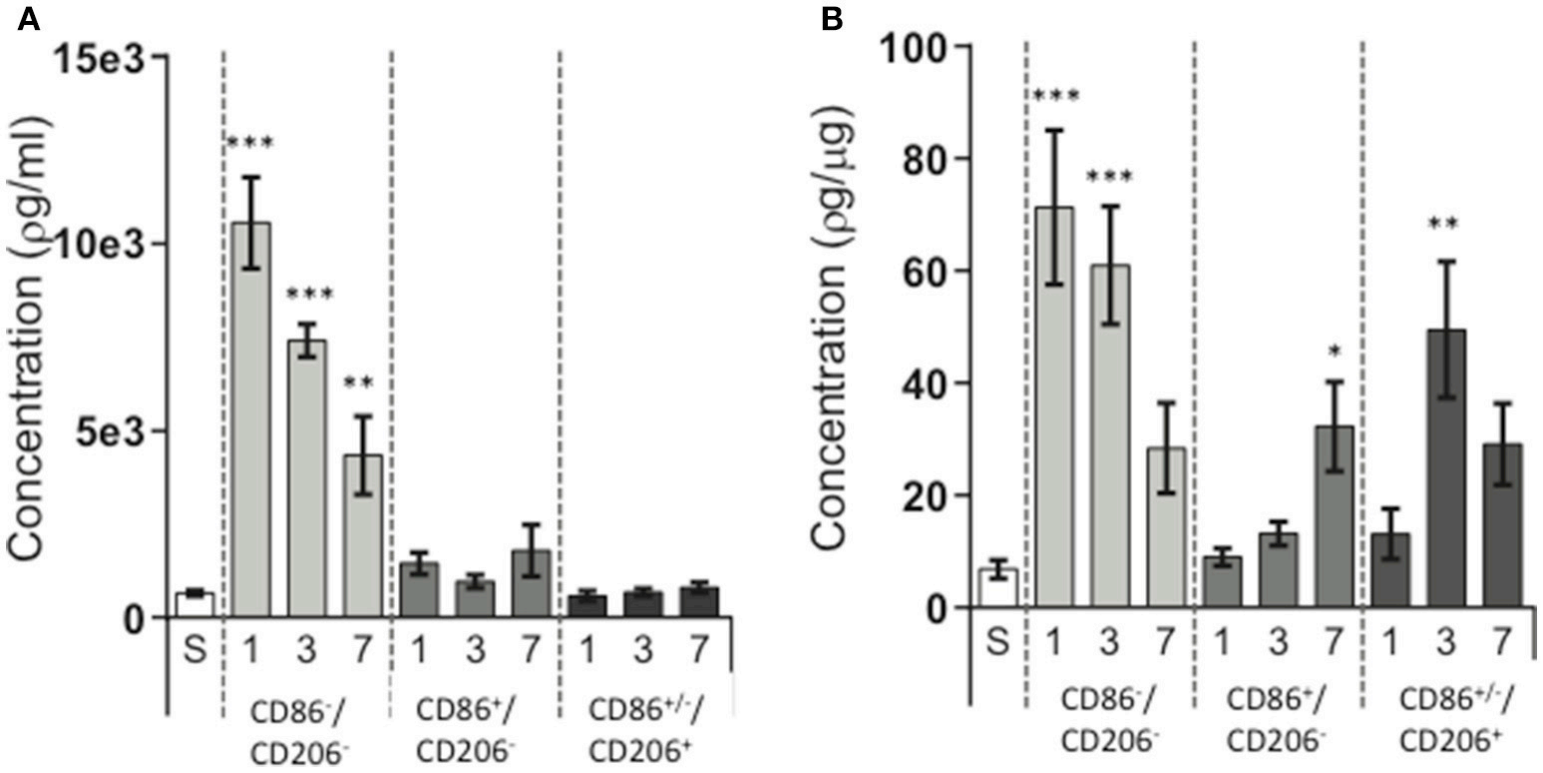
**Galectin-3 protein expression in different CD11b^**+**^ cell populations following neonatal hypoxia-ischemia**. Hypoxia-ischemia (HI) was induced in P9 mice and animals were sacrificed at 1, 3, and 7 days post HI. Brain hemispheres were processed into single cell suspension, stained with antibodies against CD11b, CD86, and CD206 and sorted by FACS. Galectin-3 protein expression was determined in lysates of sorted cell populations by ELISA. Sham operated animals (white bars), ipsilateral hemisphere in CD11b^+^CD86^−^CD206^−^ cells (light gray bars), ipsilateral hemisphere in CD11b^+^CD86^+^CD206^−^ cells (dark gray bars), ipsilateral hemisphere in CD11b^+^CD86^+^/^−^CD206^+^cells (almost black bars). Protein concentrations expressed as target protein concentration per ml **(A)** and as concentration of target protein per cell **(B)**. Statistical comparisons were made using Kruskal-Wallis followed by Dunn's multiple comparison test for each cell population vs. sham. ^*^p < 0.05, ^**^p < 0.01, ^***^p < 0.001. *n* = 6–7 animals/group.

To evaluate immunomodulatory properties of the different cell populations we investigated the protein expression of galectin-3 in lysates of FACS-sorted cell populations. Considering the marked changes in cell numbers of the different populations after HI, we analyzed both changes in protein expression in the total cell populations (pg/mL; reflecting the overall contribution of each cell population, Figure 4A) as well as corrected for protein content (pg/μg protein, Figure 4B) to represent the protein expressed per cell. Galectin-3 expression was upregulated in several cell populations (Figure 4B) with significant changes observed at 24 h (942.1%; *p* < 0.001) and at 72 h (790.9%; *p* < 0.001) in the CD11b^+^CD86^−^CD206^−^ cell population. In CD11b^+^CD86^+^CD206^−^ cells, increased galectin-3 expression was detected at 7 days (371.4%; *p* = 0.0413) and in CD11b^+^CD86^+/−^CD206^+^ cells at 72 h (623.3%; *p* = 0.0015). When investigating the protein expression in the total cell populations (pg/mL), CD11b^+^CD86^−^CD206^−^ cells were the main source of increased galectin-3 protein in the injured hemisphere at all three time points (Figure 4A).

### Immunohistochemical identification of galectin-3 in microglia with low CD86 and CD206 expression

**Figure 5:**
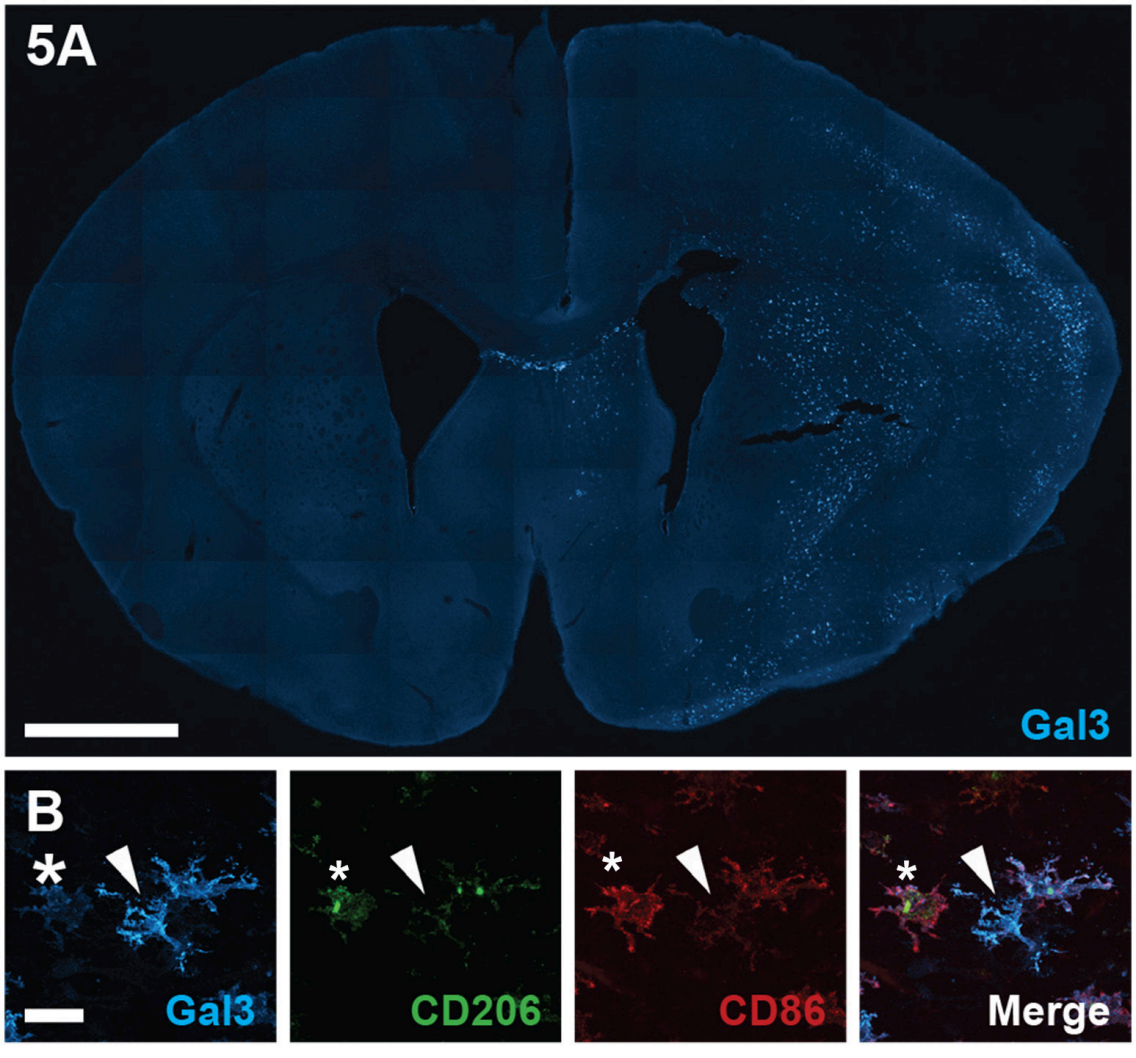
**Galectin-3 immunofluorescence staining following neonatal hypoxia-ischemia. (A)** Photomicrograph of galectin-3 immunoreactivity in a coronal section of the brain at 24 h after hypoxia-ischemia (HI), injured ipsilateral hemisphere to the right. **(B)** Representative photomicrographs of galectin-3 staining, co-labeled with CD86 and CD206 in the ipsilateral hemisphere at 24 h after HI. Star indicates cells with low galectin-3 and high CD206 and CD86 staining. White arrowhead demonstrates galectin-3 positive cell with weak CD206 and CD86 staining. Scale bars: A = 1000 μm, B = 20 μm.

To validate galectin-3 levels measured by ELISA relative to phenotypic markers *in vivo*, we conducted confocal microscopic examination on histological sections stained for galectin-3, CD86 and CD206 (Figure 5). Tile scanned photomicrographs of whole brain sections 24 h after HI revealed robust galectin-3 immunoreactivity in typically injured brain regions in the ipsilateral hemisphere, including cortical regions and the striatum, with staining absent from the contralateral hemisphere (Figure 5A). In line with our ELISA results displaying increased galectin-3 expression particularly in CD11b^+^CD86^−^CD206^−^ cells at 24 h after HI, confocal examination of individual cells in the ipsilateral cortex showed that strongly galectin-3 positive cells expressed low levels of CD86 and CD206 immunoreactivity (Figure 5B). These galectin-3 immunoreactive cells displayed amoeboid or hypertrophic but not highly ramified morphologies. In contrast, classically and alternatively polarized cells that were more strongly immunoreactive for CD86 and CD206, generally displayed a low level of galectin-3 staining (Figure 5B).

**Figure 6:**
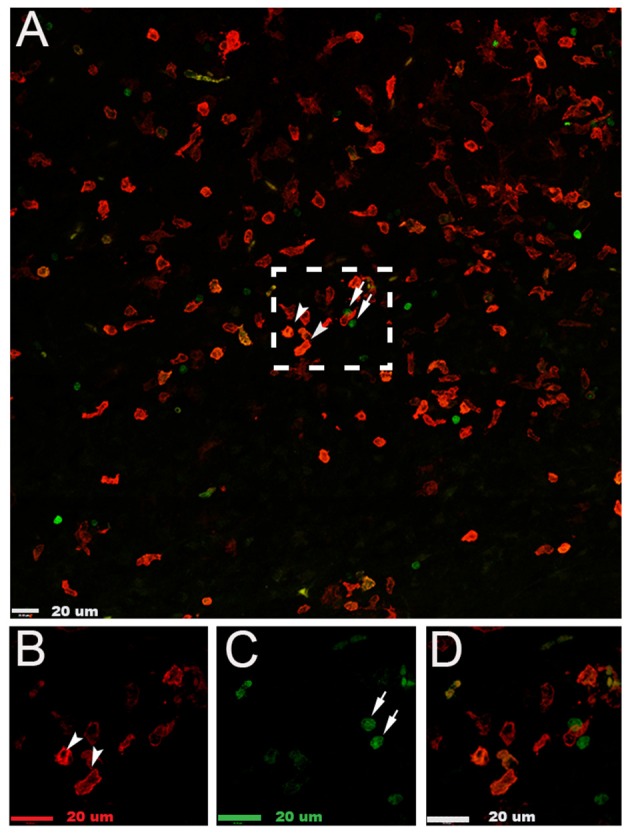
**Galectin-3 is expressed in microglia in the injured hemisphere after neonatal hypoxia-ischemia. (A)** Photomicrograph of galectin-3^+^ (red) and infiltrating myeloid EGFP^+^ (green) cells in the injured cerebral cortex 24 h after neonatal hypoxia-ischemia. **(B)** Enlarged image of galectin-3^+^ cells in box in **(A)**. **(C)** Enlarged image of myeloid EGFP^+^ (green) cells in box in **(A)**. **(D)** Enlarged image of combined staining of cells in **(A)**. White arrowheads demonstrate galectin-3 positive cells; white arrows indicate myeloid EGFP^+^ (green) cells. Scale bars = 20 μm.

By using *Lys*-EGFP-*ki* mice, that express EGFP in peripheral myeloid cells but not in microglia (Faust et al., 2000), we were able to determine that the cells expressing galectin-3 were mainly resident microglia (Figure 6).

## Discussion

In this study we investigated the applicability of using classical and alternative activation characterization of cerebral microglia/macrophages to the context of neonatal HI brain injury. We demonstrate that neonatal HI caused a rapid and transient increase in the mRNA levels of genes related to both pro- and anti-inflammatory phenotypes within the first 24 h after the initial insult. Using classical macrophage CD antigens as markers for classical and alternative activation, we identified three major CD11b^+^ cell populations in the brain by FACS analysis; cells expressing predominantly CD86 or CD206, and a cell population that lacked CD86 and CD206 expression. A dramatic expansion of the CD86 positive cells in the ipsilateral hemisphere was observed at 24 h after HI. In contrast, although increasing in real numbers, the relative proportions of CD206^+^ cells or cells lacking both CD86 and CD206 expression were reduced after injury. Using immunohistochemistry, we were able to identify Iba-1 positive cells with predominantly CD86 or CD206 staining, but the CD206 positive cells frequently co-expressed CD86 in injured areas while cells expressing CD206 only were mainly found in meninges or uninjured areas. Interestingly, protein expression of the immunomodulatory protein galectin-3 was markedly increased in cells that lacked CD86 and CD206 and this novel finding suggest that the population of these “non-polarized” cells may be important for immune responses in the injured neonatal brain.

It is well understood that macrophages and microglia can adopt distinct pro- and anti-inflammatory phenotypes in response to specific polarizing stimuli *in vitro* (Stein et al., 1992; Gordon, 2003; Chhor et al., 2013). However, the degree to which such distinct inflammatory phenotypes exist in the complex inflammatory environment of the injured CNS is less clear. Studies investigating expression of genes associated with different activation stages in the mouse middle cerebral artery occlusion (MCAO) stroke model have suggested rapid, yet transient, induction of genes associated with alternative activation followed by a later, yet sustained, induction of genes associated with classical activation (Hu et al., 2012). This data is in contrast to our observation of rapid and transient induction of genes associated with both classical (CD86, IL-6, and IL-1β), as well as alternative (IL-10, Fizz1, Arg1, and IL-10) activation followed by return to baseline expression at 24 h post HI. However, our results are in line with previous investigations in neonatal rodent HI models, which reported peak induction of IL-1β and TNF-α at 12 h after HI (Bona et al., 1999) or upregulation of IL-1β and IL-10 at 3 h (Bonestroo et al., 2013), suggesting that the inflammatory response to injury differ in the neonatal brain compared to the adult.

Although useful in supplying information about the general inflammatory response in the brain, examination of whole hemispheric gene expression fails to address the phenotypic composition of the microglia/macrophage pool. In order to address these issues we employed FACS to characterize and isolate CD11b^+^ cells based on their differential expression of CD86 and CD206, both well characterized markers of different activation phenotypes *in vitro* (Stein et al., 1992; Chhor et al., 2013). This approach allowed identification of three major CD11b^+^ cell populations: the CD86^−^CD206^−^ and the CD86^+^CD206^−^ populations were largely homogenous, while the cells positive for CD206 were more heterogeneous. Based on previously well-established gating strategies (Bedi et al., 2013), these cells were defined by their expression of CD206 but included cells also expressing CD86 antigens. Such findings are not without precedent in the adult brain: flow cytometry studies have displayed co-expression of CD206 and the pro-inflammatory marker major histocompatibility complex class II (MHCII) in the intact CNS (Li et al., 2014), and CD206^+^/FcγRII/III^+^cells have been detected in a mouse model of traumatic brain injury (Bedi et al., 2013). In addition, immunohistological interrogation of HLA-DR^+^ foamy macrophages in active human multiple sclerosis lesions indicated that 70% display co-immunoreactivity for CD206 and the pro-inflammatory marker CD40 (Vogel et al., 2013). Further, our previous *in vitro* work with microglia using these markers supports the complexity of our *in vivo* observations. We observed that even using pro-typical inducers of classic and alternative activation (LPS & IL-4), the expression of CD86 and CD206 could not be used to discriminate between these two polarization states at all time points (from 4 to 72 h post-exposure) (Chhor et al., 2013). Interestingly, using inducer-switching experiments, we also demonstrated that expression of these two markers is significantly altered by previous activation suggesting that over time *in vivo* microglia might be primed by the complex milieu of cytokines and chemokine in the local micro-environment modulating the expression patterns observed.

In our model, immunohistochemistry revealed that the cell populations expressing CD206 only or CD206 as well as CD86 were distinctly different regarding location. We identified a small population of “pure” CD206^+^ cells, which was frequently observed in the meninges. These cells have previously been identified and described as “non-parenchymal macrophages” (Galea et al., 2005). Further, we found single CD206-expressing cells in the contralateral hemisphere, as well as in sham-operated animals, consistent with the hypothesis that some microglia possess an alternative activation-like phenotype in the intact CNS (Ponomarev et al., 2007). The presence of a “mixed” population expressing both CD206 and CD86, particularly in the cerebral white matter, as well as a large population of seemingly non-polarized cells (lacking both CD86 and CD206 expression) also illustrates the complexity of the cellular response in the brain *in vivo*. Taken together, our data suggest that the traditional classic vs. alternative activation classification scheme oversimplifies the concept of distinct inflammatory cell phenotypes in the brain *in vivo*, which is also being recognized by the macrophage research field (Murray et al., 2014; Nahrendorf and Swirski, 2016).

Within the first 24 h after HI all three cell populations increased in the injured hemisphere with the largest expansion seen for cells expressing CD86. This population dominated in the HI hemisphere, and although both the total number and the proportion of these cells steadily decreased with time after injury the total number of cells was still elevated at 7 days in the ipsilateral compared to contralateral hemisphere. Concomitant with this, cell populations expressing CD206 or lacking both CD86 and CD206 constituted a relatively smaller proportion of cells for several days after HI, despite their increase in absolute numbers. Studies of adult spinal cord injury (Kigerl et al., 2009) and MCAO (Hu et al., 2012) have suggested a gradual increase in both classically and alternatively activated microglia in the first days after insult, with the presence of classically activated microglia continuing to increase up to 28 days, whilst the alternative activation response appeared transient with numbers peaking at 5–7 days and decreasing gradually thereafter. Thus, our findings suggest differences in neonatal and adult CNS immune responses to injury.

A novel finding in this study is that the cell population lacking CD86 and CD206 expression was the major contributor of galectin-3 protein expression after HI. In support, immunohistochemical analysis showed that cells with the highest galectin-3 staining intensity expressed little CD86 or CD206 activation markers, yet exhibited the amoeboid appearance associated with activated phenotypes. These results further emphasize the inadequacy of using CD86 and CD206 as markers of microglia/macrophage activation. Galectin-3 is an immunomodulatory factor that is involved in activation and polarization of inflammatory cells, probably by inducing pro-inflammatory cytokines (Jeng et al., 1994) and the release of oxygen free radicals (Karlsson et al., 1998). Galectin-3 expression in microglia is increased in response to the pro-typical alternative activation inducer IL-4 *in vitro* (Chhor et al., 2013) and it has been suggested that galectin-3 is essential for polarization through a feed-back loop initiated by IL-4 (MacKinnon et al., 2008). Our results demonstrate galectin-3 to be strongly upregulated in response to injury. It is presently unknown to what extent peripheral monocytes/macrophages may contribute to cellular immune responses after neonatal HI (Mallard and Vexler, 2015). Our data suggest that there is some degree of infiltration of myeloid cells as indicated by the presence of EGFP positive cells in the brain after HI. However, few of the EGFP positive cells expressed galectin-3, suggesting that galectin-3 is mainly expressed by microglia. Potentially, these cells may be important in attracting and priming cells to additional stimuli and thus modulating the inflammatory response, which appears to be critical for development of brain injury according to previous work (Doverhag et al., 2010).

## Conclusions

We have shown a dynamic expression pattern of pro- and anti-inflammatory mRNA in the brain after neonatal HI. CD11b^+^ cells that express the CD86 surface antigen, which has been associated with classical activation of macrophages, dominated in the injured brain during the first days after HI. Cells expressing antigen associated with alternative activation (CD206) demonstrated a similar expansion over time, but with a smaller magnitude of the increase resulting in a relative suppression of this cell type. The CD206 expressing cells were heterogeneous and there were cells expressing CD206 as well as CD86, thus illustrating the complexity of the cellular immune response in the brain after injury. A novel finding is that a large population of CD11b^+^ cells in the brain after neonatal HI is “non-polarized” with regard to classical activation markers CD86 and CD206, but express high amounts of the immunomodulatory factor galectin-3. We speculate that this population may be important for attracting cells to injured areas and could be involved in modulation of the post-injury inflammatory response.

## Ethics statement

All animal experiments were conducted in accordance with regulations and general guidelines of the Swedish Board of Agriculture (Sweden) and were approved by the regional Gothenburg Animal Ethics Board (No. 374/09 and 139/13).

## Author contributions

Conception and design: NHE, KB, KS, and CM. Acquisition of data and analysis: NHE, PS, BF, WW, MB, SN, and PS. Interpretation of data: NHE, PS, KS, and CM. Drafting and critically revised the manuscript for important intellectual content: NHE, PS, BF, WW, MB, PG, HH, KB, KS, and CM.

## Funding

The studies were supported by funding from the Swedish Research Council (VR2012–2992, CM), Government grant in Public Health Service at the Sahlgrenska University Hospital (ALFGBG-142881, CM, KS), European Union grant FP7, (Neurobid, HEALTHF2–2009–241778, CM), the Leducq foundation (DSRR_P34404, CM, HH), Åhlén Foundation (CM), Olle Engkvist Byggmästare (CM), the Swedish Brain Foundation (FO2013–095, CM), Torsten Söderberg (M98/15, CM), Wilhelm and Martina Lundgren Scientific Foundation (KS), Linnéa and Josef Carlsson Foundation (KS).), the Swedish Childhood Cancer Foundation (MB), the Frimurare Barnhus Foundation (MB) and Lions Cancer Foundation (MB).

### Conflict of interest statement

The authors declare that the research was conducted in the absence of any commercial or financial relationships that could be construed as a potential conflict of interest. The reviewer RM and handling Editor declared their shared affiliation, and the handling Editor states that the process nevertheless met the standards of a fair and objective review.

## Abbreviations

ANOVA: analysis of variance
ARG1: arginase 1
BSA: bovine serum albumin
CD: cluster of differentiation
CNS: central nervous system
Contra: contralateral hemisphere
COX2: cyclooxygenase 2
CT: threshold cycle
EDTA: ethylenediaminetetraacetic acid
EGFP: enhanced green fluorescent protein
ELISA: enzyme-linked immunosorbent assay
FACS: fluorescence-activated cell sorting
FSC: forward scatter
FIZZ1: resistin-like molecule alpha1
GAPDH: glyceraldehyde-3-phosphate dehydrogenase
HBSS: Hank's Balanced Salt Solution
HI: hypoxia-ischemia
IBA1: Ionized calcium binding adapter molecule 1
IL: interleukin
iNOS: inducible nitric oxide synthase
IPSI: ipsilateral hemisphere
MCP-1: monocyte chemoattractant protein-1
MCAO: middle cerebral arterial occlusion
MHC II: major histocompatibility complex, class II
P: postnatal day
PBS: phosphate-buffered saline
RT: room temperature
SSC: side scatter
TNF-α: tumor necrosis factor alpha.
