# Supplementary material for: Temporal Characterization of Microglia/Macrophage Phenotypes in a Mouse Model of Neonatal Hypoxic-Ischemic Brain Injury
Source: Front Cell Neurosci. 2016 Dec 15;10:286. doi: 10.3389/fncel.2016.00286 (PMC5156678; doi:10.3389/fncel.2016.00286)
Supplement: Supplementary file 1 [file Table1.DOCX]

**Supplementary table 1. Two-way Anova table for gene expression analysis**

| **Gene** | **ss** | **df** | **MS** | **F(DFn, DFd)** | **P value** |
| --- | --- | --- | --- | --- | --- |
| **CD86** | | | | | |
| Interaction | 2,485 | 3 | 0,8284 | F (3, 84) = 25,84 | P < 0,0001 |
| Time | 3,040 | 3 | 1,013 | F (3, 84) = 31,61 | P < 0,0001 |
| Hemisphere | 1,707 | 1 | 1,707 | F (1, 84) = 53,25 | P < 0,0001 |
| **IL-6** | | | | | |
| Interaction | 2,485 | 3 | 0,8284 | F (3, 84) = 25,84 | P < 0,0001 |
| Time | 3,040 | 3 | 1,013 | F (3, 84) = 31,61 | P < 0,0001 |
| Hemisphere | 1,707 | 1 | 1,707 | F (1, 84) = 53,25 | P < 0,0001 |
| **IL-1b** | | | | | |
| Interaction | 8827 | 3 | 2942 | F (3, 82) = 8,415 | P < 0,0001 |
| Time | 9039 | 3 | 3013 | F (3, 82) = 8,617 | P < 0,0001 |
| Hemisphere | 3032 | 1 | 3032 | F (1, 82) = 8,671 | P = 0,0042 |
| **Cox2** | | | | | |
| Interaction | 13,04 | 3 | 4,345 | F (3, 84) = 3,612 | P = 0,0165 |
| Time | 29,47 | 3 | 9,823 | F (3, 84) = 8,165 | P < 0,0001 |
| Hemisphere | 0,9941 | 1 | 0,9941 | F (1, 84) = 0,8263 | P = 0,3659 |
| **iNOS** | | | | | |
| Interaction | 0,1923 | 3 | 0,06411 | F (3, 84) = 0,6961 | P = 0,5570 |
| Time | 0,3500 | 3 | 0,1167 | F (3, 84) = 1,267 | P = 0,2910 |
| Hemisphere | 0,5684 | 1 | 0,5684 | F (1, 84) = 6,172 | P = 0,0150 |
| **CD206** | | | | | |
| Interaction | 0,004200 | 3 | 0,001400 | F (3, 84) = 0,0298 | P = 0,9930 |
| Time | 0,6691 | 3 | 0,2230 | F (3, 84) = 4,748 | P = 0,0042 |
| Hemisphere | 0,0002923 | 1 | 0,0002923 | F (1, 84) = 0,0062 | P = 0,9373 |
| **IL-10** | | | | | |
| Interaction | 0,8676 | 3 | 0,2892 | F (3, 82) = 29,27 | P < 0,0001 |
| Time | 1,039 | 3 | 0,3464 | F (3, 82) = 35,07 | P < 0,0001 |
| Hemisphere | 0,2329 | 1 | 0,2329 | F (1, 82) = 23,58 | P < 0,0001 |
| **Fizz 1** | | | | | |
| Interaction | 3,512 | 3 | 1,171 | F (3, 83) = 7,824 | P = 0,0001 |
| Time | 3,174 | 3 | 1,058 | F (3, 83) = 7,071 | P = 0,0003 |
| Hemisphere | 0,1938 | 1 | 0,1938 | F (1, 83) = 1,295 | P = 0,2584 |
| **Arginase 1** | | | | | |
| Interaction | 2,941 | 3 | 0,9803 | F (3, 84) = 24,27 | P < 0,0001 |
| Time | 5,443 | 3 | 1,814 | F (3, 84) = 44,92 | P < 0,0001 |
| Hemisphere | 1,595 | 1 | 1,595 | F (1, 84) = 39,48 | P < 0,0001 |
| **Galectin 3** | | | | | |
| Interaction | 0,2300 | 3 | 0,07667 | F (3, 84) = 21,35 | P < 0,0001 |
| Time | 0,3157 | 3 | 0,1052 | F (3, 84) = 29,31 | P < 0,0001 |
| Hemisphere | 0,3071 | 1 | 0,3071 | F (1, 84) = 85,53 | P < 0,0001 |
