# Supplementary material for: Temporal Characterization of Microglia/Macrophage Phenotypes in a Mouse Model of Neonatal Hypoxic-Ischemic Brain Injury
Source: Front Cell Neurosci. 2016 Dec 15;10:286. doi: 10.3389/fncel.2016.00286 (PMC5156678; doi:10.3389/fncel.2016.00286)
Supplement: Supplementary file 2 [file Table2.DOCX]

**Supplementary table 2. Two-way Anova table for cell count analysis**

| **Cell type** | **ss** | **df** | **MS** | **F(DFn, DFd)** | **P value** |
| --- | --- | --- | --- | --- | --- |
| **CD11b^+^** | | | | | |
| Interaction | 9,656e+011 | 2 | 4,828e+011 | F (2, 29) = 73,48 | P < 0,0001 |
| Time | 1,171e+012 | 2 | 5,857e+011 | F (2, 29) = 89,13 | P < 0,0001 |
| Hemisphere | 1,946e+012 | 1 | 1,946e+012 | F (1, 29) = 296,2 | P < 0,0001 |
| **CD11b^+^CD86^-^CD206^-^** | | | | | |
| Interaction | 6,648e+010 | 2 | 3,324e+010 | F (2, 29) = 11,57 | P = 0,0002 |
| Time | 1,362e+011 | 2 | 6,810e+010 | F (2, 29) = 23,70 | P < 0,0001 |
| Hemisphere | 2,506e+011 | 1 | 2,506e+011 | F (1, 29) = 87,23 | P < 0,0001 |
| **CD11b^+^CD86^+^CD206^-^** | | | | | |
| Interaction | 4,378e+011 | 2 | 2,189e+011 | F (2, 29) = 75,27 | P < 0,0001 |
| Time | 4,701e+011 | 2 | 2,350e+011 | F (2, 29) = 80,83 | P < 0,0001 |
| Hemisphere | 6,806e+011 | 1 | 6,806e+011 | F (1, 29) = 234,0 | P < 0,0001 |
| **CD11b^+^ CD86^+/-^CD206^+^** | | | | | |
| Interaction | 4,360e+009 | 2 | 2,180e+009 | F (2, 29) = 69,78 | P < 0,0001 |
| Time | 4,178e+009 | 2 | 2,089e+009 | F (2, 29) = 66,87 | P < 0,0001 |
| Hemisphere | 4,709e+009 | 1 | 4,709e+009 | F (1, 29) = 150,7 | P < 0,0001 |
