# Supplementary material for: Temporal Characterization of Microglia/Macrophage Phenotypes in a Mouse Model of Neonatal Hypoxic-Ischemic Brain Injury
Source: Front Cell Neurosci. 2016 Dec 15;10:286. doi: 10.3389/fncel.2016.00286 (PMC5156678; doi:10.3389/fncel.2016.00286)
Supplement: Supplementary file 3 [file Table3.DOCX]

**Supplementary table 3. Two-way Anova table for % cells of total CD11b+ population**

| **Cell type** | **ss** | **df** | **MS** | **F(DFn, DFd)** | **P value** |
| --- | --- | --- | --- | --- | --- |
| **CD11b^+^CD86^-^CD206^-^** | | | | | |
| Interaction | 1331 | 2 | 665,3 | F (2, 32) = 57,97 | P < 0,0001 |
| Time | 1010 | 2 | 505,0 | F (2, 32) = 44,01 | P < 0,0001 |
| Hemisphere | 8290 | 1 | 8290 | F (1, 32) = 722,4 | P < 0,0001 |
| **CD11b^+^CD86^+^CD206^-^** | | | | | |
| Interaction | 4,378e+011 | 2 | 2,189e+011 | F (2, 29) = 75,27 | P < 0,0001 |
| Time | 4,701e+011 | 2 | 2,350e+011 | F (2, 29) = 80,83 | P < 0,0001 |
| Hemisphere | 6,806e+011 | 1 | 6,806e+011 | F (1, 29) = 234,0 | P < 0,0001 |
| **CD11b^+^ CD86^+/-^CD206^+^** | | | | | |
| Interaction | 32,30 | 2 | 16,15 | F (2, 32) = 8,168 | P = 0,0014 |
| Time | 23,67 | 2 | 11,83 | F (2, 32) = 5,985 | P = 0,0062 |
| Hemisphere | 42,62 | 1 | 42,62 | F (1, 32) = 21,56 | P < 0,0001 |
